# Triggered dynamics in a model of different fault creep regimes

**DOI:** 10.1038/srep05401

**Published:** 2014-06-23

**Authors:** Srđan Kostić, Igor Franović, Matjaž Perc, Nebojša Vasović, Kristina Todorović

**Affiliations:** 1 Department of Geology, University of Belgrade, Faculty of Mining and Geology, Serbia; 2 Scientific Computing Lab., Institute of Physics, University of Belgrade, PO Box 68, 11080 Beograd-Zemun, Serbia; 3 Department of Physics, Faculty of Natural Sciences and Mathematics, University of Maribor, Slovenia; 4 Department of Applied Mathematics, University of Belgrade, Faculty of Mining and Geology, Serbia; 5 Department of Physics and Mathematics, University of Belgrade, Faculty of Pharmacy, Serbia; 6 Faculty of Science, King Abdulaziz University, Jeddah, Saudi Arabia

## Abstract

The study is focused on the effect of transient external force induced by a passing seismic wave on fault motion in different creep regimes. Displacement along the fault is represented by the movement of a spring-block model, whereby the uniform and oscillatory motion correspond to the fault dynamics in post-seismic and inter-seismic creep regime, respectively. The effect of the external force is introduced as a change of block acceleration in the form of a sine wave scaled by an exponential pulse. Model dynamics is examined for variable parameters of the induced acceleration changes in reference to periodic oscillations of the unperturbed system above the supercritical Hopf bifurcation curve. The analysis indicates the occurrence of weak irregular oscillations if external force acts in the post-seismic creep regime. When fault motion is exposed to external force in the inter-seismic creep regime, one finds the transition to quasiperiodic- or chaos-like motion, which we attribute to the precursory creep regime and seismic motion, respectively. If the triggered acceleration changes are of longer duration, a reverse transition from inter-seismic to post-seismic creep regime is detected on a larger time scale.

Fault creep investigation lies in the focus of earthquake hazard assessment, since creeping is often regarded as a possible indication of a pending earthquake magnitude^1^. Although fault creep is typically slow (<30 mm/yr), the probability of generating large earthquakes along the creeping faults is generally lower, primarily because the slip area in the future earthquake is smaller along the fault that creeps^2^. An alternative explanation of such reduced earthquake possibility along the creeping faults lies in the fact that the slow slippage may partially relieve stress buildup along the faults, thereby decreasing the magnitude of the next seismic event.

Creep motion refers to aseismic slip lasting over several decades between the major earthquakes^3^. According to the temporal distance between the preceding and the pending earthquake, i.e. depending on the phase of an earthquake cycle, several creep regimes are typically observed. Post-seismic creep regime is observed immediately after the seismic motion ceases and is commonly considered as a short period of a few years or decades after the last earthquake. Nevertheless, inter-seismic period is regarded as a period of slow accumulation of elastic strain that coincides with frictional locking of a fault between earthquakes, which may last from decades to thousands of years^4^. The third stage of fault creeping is denoted as a precursory creep regime, typically encountered before a seismic event. Transitions between the different creep regimes occur due to increased accumulation of stress along the fault, eventually leading to the onset of seismic motion. Another reason for switching between the different stages of fault slip lies in the external impact of near or distant earthquakes, which primarily trigger the static and dynamic stress variations along the fault^5^. Instantaneous triggering is typically observed when the external force originated from the passing seismic wave acts along the fault which approaches the end of an earthquake cycle^6^,^7^,^8^. Apart from this instantaneous triggering effect, some locations experience a delayed onset of motion along the fault, which occurs due to a variety of time-dependent stress transfer mechanisms, including the viscous relaxation, poroelastic rebound and afterslip, or could be caused by reductions in fault friction, as predicted by the rate and state constitutive relations^7^,^9^. These dynamic stress changes may also be responsible for the occurrence of an aftershock sequence just after the mainshock^10^. All these effects could promote a transition between the creep regimes, which may sometimes involve the skipping of the succeeding stage of fault motion, leading eventually to the earthquake nucleation.

Issue of dynamic triggering has been the subject of an extensive research, focusing on one hand on the analysis of the induced seismic events in situ, and on the investigation of the dynamics of spring-slider models from the aspect of a possible advance of an earthquake cycle, on the other hand. The former relies on the recorded evidence of earthquakes which occurred after the large seismic events, including the ones detected in Yellowstone National Park after the 1992 M7.3 Landers earthquake^11^, the seismic events in southeastern California after the 2002 M7.9 Denali earthquake^12^ or the earthquakes in Greece following the 1999 M7.4 Izmit earthquake^6^. As far as the analysis of phenomenological models is concerned, Gomberg et al.^13^ have shown using a simple massless spring-slider system that the transients hasten the time of earthquakes that ultimately would have happened due to the constant background loading alone. Perfettini et al.^14^ extended the work of Gomberg et al.^13^ by studying the effect of pulses and wave packets in a 2-D continuous quasi-dynamic fault model coupled with Dieterich-Ruina friction law under the variations of both shear and normal stress. Their analysis indicates that dynamic triggering by seismic waves can occur only in the areas of higher pore pressures or along the faults at the end of their earthquake cycle. Du et al.^15^ used the same sine wave transient load, as in Gomberg et al.^13^ and Perfettini et al.^14^, and applied it as a forcing function to the spring-slider system. It has been found that certain types of transient loads can trigger the next anticipated creep events, which then occur either shortly after the transient load ends or with a time delay. This has also been shown by Belardinelli et al.^16^, who analyzed a spring-slider model including inertia, in order to examine the dependence of triggering delays on different system conditions and constitutive parameters. According to their results, the static stress change can advance as well as delay an induced instability depending on its sign, whereas the dynamic stress pulse can only promote a nearly instantaneous failure, though under condition that its amplitude is positive and large enough with respect to the direct effect of friction.

However, despite the notable amount of research on the effects of dynamic triggering on the motion of spring-slider models and in situ, there is no evidence whether the passing seismic wave can induce transitions between the different slip regimes, which is the issue addressed in detail in present paper. The performed analysis is based on the dynamics of spring-slider model coupled with the Dieterich-Ruina friction law, which is assumed to qualitatively describe aseismic displacement along the fault. The impact of a passing seismic wave is mathematically described by a sine wave scaled by an exponential pulse, as already suggested in Gomberg et al.^13^ and Perfettini et al.^14^. However, in contrast to the previous studies, we assume that the acceleration changes of the block attain such a wave form.

It is important to underline that the analysis of the mono-block model, as a crude picture of the fault slip, is limited in two ways. On one hand, there is a lack of knowledge on the initial state of the fault, both in terms of stress state and the geometrical features, whereas on the other hand, there is insufficient understanding of the governing laws that describe the dissipative physico-chemical mechanisms presumably occurring during a fault motion^17^,^18^,^19^. However, in the present study we deliberately adopt the simplest model in order to qualitatively understand the concept of dynamic triggering, without considering the possible complications that arise from the spatial heterogeneities and geometrical complexity of fault structures handled in the extended fault models.

One should be aware of the fact that the performed research is purely phenomenological, in a sense that the intention is to capture the main mechanisms of fault dynamics under the effect of an external force induced by a passing seismic wave, without setting the goal to solve the complex geological and tectonic relations in the Earth's crust. The objective of the analysis is not to identify the nature of the triggered seismicity, but rather to examine whether it is possible to generate the transition between the different slip regimes and, eventually, induce a seismic motion, only by perturbing the block's acceleration, reflecting, in that way, the effect of a transient seismic wave. In effect, the presented analysis is intended to determine which creep regime is most sensitive to external perturbation.

## Results

In the present paper, we start from the assumption that motion along a single fault could be described by a mono-block spring-slider model coupled with the Dieterich-Ruina rate-and state-dependent friction law, as first proposed in Erickson et al.^20^: 
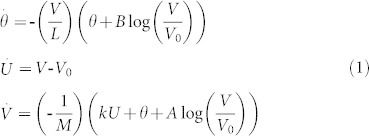
where parameter *M* denotes the mass of the block and the spring stiffness *k* corresponds to the linear elastic properties of the rock mass surrounding the fault^21^. This physical system is analogous to a single fault patch of fixed dimensions that ruptures in an elastic medium^22^. According to Dieterich and Kilgore^23^ parameter *L* corresponds to the critical sliding distance necessary to replace the population of asperity contacts. The parameters *A* and *B* are empirical constants, which depend on the properties of material. Ruina^24^ suggested that parameter *A* quantifies the direct velocity dependence (“direct effect”), while *(A − B)* is a measure of the steady-state velocity dependence. For convenience, system (1) is non-dimensionalized by defining the new variables *θ′*, *V′*, *U′* and *t′* in the following way: *θ* = *Aθ′*, *V* = *V_0_V′*, *U* = *LU′*, *t* = *(L/V_0_)t′*. The nondimensional system, once we return to the original notation, takes the following form: 
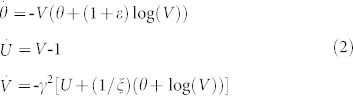
where *ε* = (*B* − *A*)/*A* measures the sensitivity of velocity relaxation, *ξ* = (*kL*)/*A* is the nondimensional spring constant, and 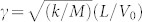 is the nondimensional frequency^20^.

System (2) represents a fault slip model coupled with the Dieterich-Ruina friction law, so unless it is perturbed, it only describes different types of creep regimes. The dynamics of system (2) for different values of control parameters (*ε*, *ξ*, *γ*) is analyzed in section Methods.

Our focus lies with the analysis of the dynamics of system (2) under the effect of external periodic force, which is induced by a passing seismic wave and which directly affects the acceleration of the block in a form of seismic train oscillation: 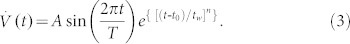
In the above expression, *A* denotes the maximum amplitude of the induced change, *T* is the wave period, *t_0_* is the moment for which the perturbation amplitude reaches its maximum value, *t_w_* is the half width of the induced perturbation, and *n* represents an integer that controls the rise-time^14^. This acceleration change consists of a pulse like envelope inside which oscillations of period *T* occur. In other words, the effect of a transient external force is simulated using a sine wave of period *T*, scaled by an exponential pulse with amplitude *A* and width 2*t_w_*. By introducing the transient wave (3) in the observed system (2) we obtain: 
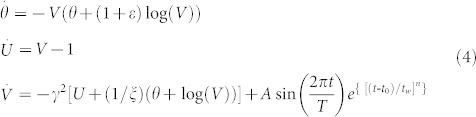
The effect of different types of external force is reflected in dependence on period *T*. In terms of real earthquakes, more rapidly varying (short-period) transient changes of block acceleration could correspond to the effect of closer, smaller earthquakes, while more slowly varying (long-period) transient changes might describe larger, more remote earthquakes^13^.

Note that we do not consider the effect of a passing seismic wave in reference to the phase of the seismic cycle, simply because our model, for the particular parameter values, does not exhibit stick-slip motion, which is regarded as a typical seismic movement. On the contrary, the adopted fault slip model (2) describes creeping along faults, with an earthquake arising once the block starts to move in an irregular chaotic-like fashion. This way, we are able to observe the possible transitions between the different aseismic and seismic slip regimes under the impact of a triggering external force produced by the passing seismic wave from near or distant earthquakes. Concerning this, throughout the study, the parameters of perturbation are set as follows. Analysis is performed for the transient acceleration changes of different half-duration 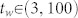 and different period 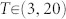, in comparison to the frequency of periodic oscillation of the unperturbed system (2) just above the bifurcation curve (*T_0_* ≈ 12). Regarding the time for which the amplitude of the induced change reaches its maximum value, we assume a value of *t_0_* = 1000, which is sufficiently larger than the time required for the dynamics of the unperturbed system (2) to stabilize on the limit cycle just above the bifurcation curve. Value of parameter *n*, which controls the rise-time, is set to 16, as already proposed in Perfettini et al.^14^.

The amplitude of the acceleration change, *A* = 0.6, is selected by drawing comparison to the amplitude of oscillations exhibited by system (2) just above the bifurcation curve. In the present paper, we consider the amplitudes that are equal, slightly larger or smaller (two times in both cases) than the amplitude of periodic oscillation of the unperturbed system. Note that the oscillations of the unperturbed system (2) just above the bifurcation curve do not represent an example of a seismic cycle, but only another mode of creep motion. Hence, the amplitude of oscillatory motion does not have an immediate physical interpretation and we consider it unrelated to the seismic events.

### Transient triggering in the post-seismic creep regime

Under the assumption that a passing seismic wave acts along a fault in the post-seismic creep regime, analysis of block's dynamics is performed for the parameter values below the bifurcation curve (*ε* = 0.2, *ξ* = 0.5, *γ* = 0.8), where the motion of the block is uniform.

For the brief short-period transient changes, represented by the case *t_w_* = 3 and *T* = 3, the induced dynamics of the perturbed system (4) shows only a somewhat higher sharp first amplitude response, without irregular oscillations. Qualitatively similar effect is found for the brief long-period acceleration changes. Nevertheless, the transient switch to periodic oscillations is observed for the longer high-frequent changes, which can be regarded as a temporary transition to the inter-seismic creep regime.

**Figure 1:**
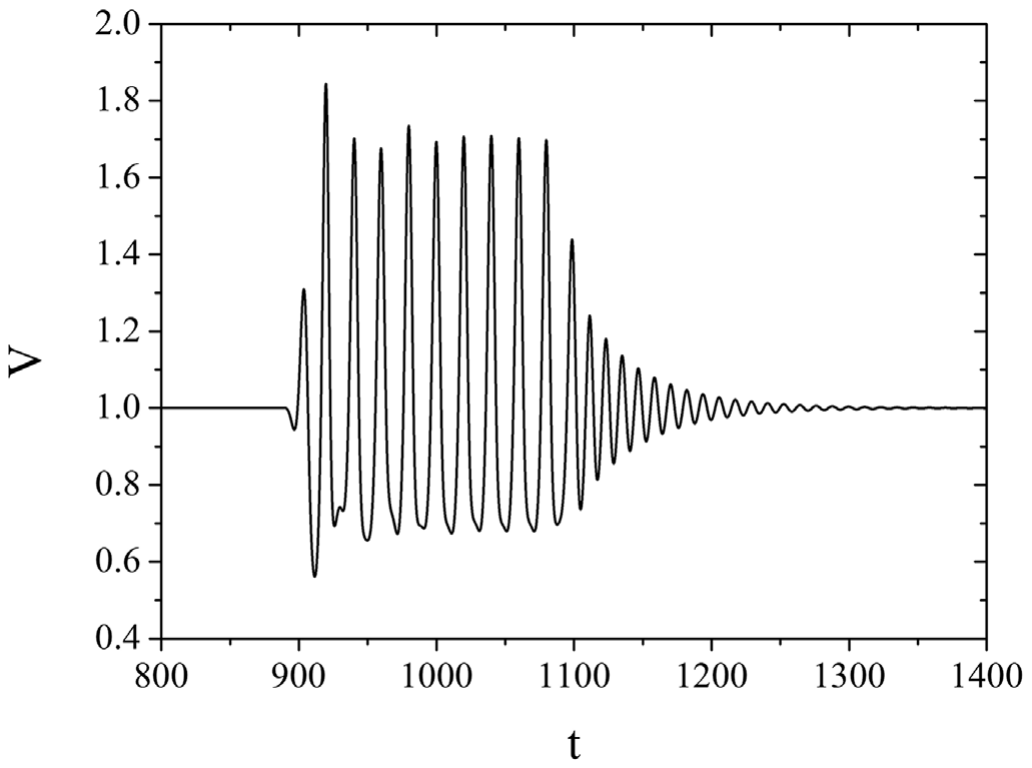
Evolution of variable *V* for *ε* = 0.2, *ξ* = 0.5 and *γ* = 0.8 under the influence of the low-frequent acceleration change (*T* = 20) with longer duration (*t_w_* = 100). The remaining parameter values are: *A* = 0.6, *t_0_* = 1000 and *n* = 16. Note that the velocity and time are considered as non-dimensional quantities.

**Figure 2:**
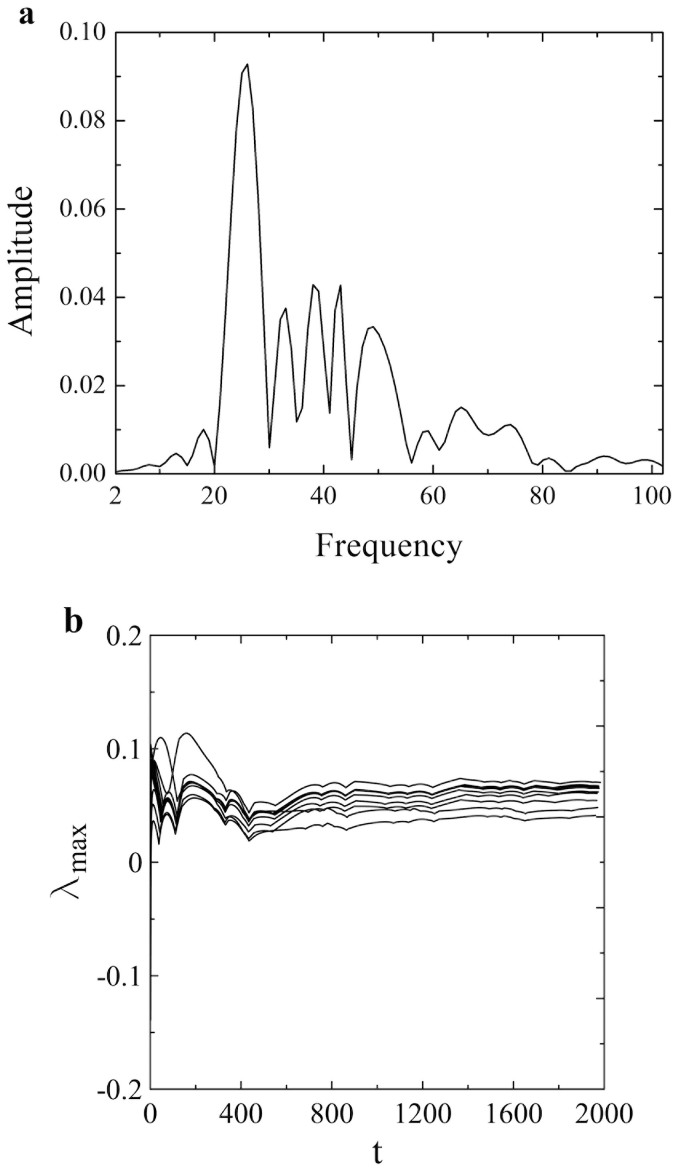
(a) The continuous broadband noise in the Fourier power spectrum for the time series in Figure 1 indicates that the system exhibits a relatively weak chaotic behavior. (b) Calculation of the maximal Lyapunov exponent involves the additional averaging over a set of different initial conditions, whereby *θ*, *U*, *V* belong to the respective ranges 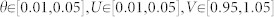. The results are obtained by adapting the method of Wolf et al.^26^. Maximal Lyapunov exponents are seen to converge well to the positive values in the range *λ_max_* = 4.1 − 7.4 × 10^−2^. Note that time *t* is expressed in the units of iteration steps.

For the longer low-frequent acceleration changes, represented by the case *t_w_* = 100 and *T* = 20, one encounters weakly irregular oscillations, cf. Figure 1. The irregular character of oscillations is corroborated by the broadband noise in the corresponding Fourier power spectrum (Figure 2a). Given the irregularity of the observed motion, this type of triggered effect could be considered an instance of immediate (instantaneous) earthquake triggering. However, note that the question of qualifying certain transient motion as transient chaos (or, at least, chaos-like behavior) should in principle be treated by determining the finite-time Lyapunov exponent^25^. Here we determine the largest Lyapunov exponent for the time series showing the sufficiently long transient according to the general method of Wolf et al.^26^, whereby the standard procedure is complemented by carrying out the additional averaging over a set of different initial conditions (Figure 2b). The convergence condition is satisfied in a sense that the approximately stationary values of the largest Lyapunov exponents are reached on the time scale (*t* ≈ 30) significantly smaller than the transient length (*t* ≈ 100). The maximal Lyapunov exponents determined for the different initial conditions are found to converge well to positive values in the range 4.1 − 7.4 × 10^−2^.

Qualitatively similar phenomena are observed in case of larger perturbation amplitudes (*A* = 1.2), whereas the impact of smaller amplitudes (*A* = 0.3) is marginal.

### Transient triggering in the inter-seismic creep regime

In this case, the analysis is performed for the parameter values just above the bifurcation curve (*ε* = 0.3, *ξ* = 0.5, *γ* = 0.8), where the motion of the block is oscillatory.

In case of a brief short-period change, for *t_w_* = 3 and *T* = 3, the system (4) reacts through a sharp first response with a slightly higher amplitude, then displaying a slower convergence to the limit cycle relative to the unperturbed motion. Note that this is similar to the case where the system (2) lies in the steady state post-seismic regime. The qualitatively analogous effect occurs for the long-period transient changes (*T* = 20) of short duration.

**Figure 3:**
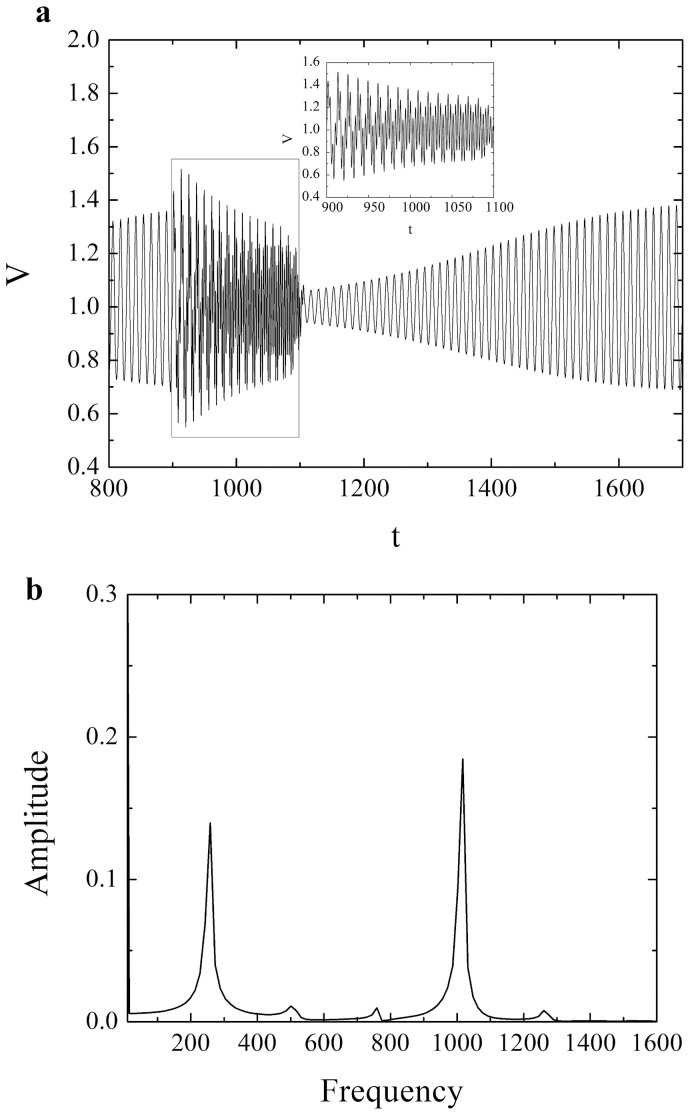
(a) Evolution of variable *V* for *ε* = 0.3, *ξ* = 0.5 and *γ* = 0.8 induced by the short-period acceleration change (*T* = 3) with longer duration (*t_w_* = 100). The remaining parameters are set to: *A* = 0.6, *t_0_* = 1000 and *n* = 16. The rectangular frame indicates the quasiperiodic-like transient part of the time series, whose blowup is displayed in the inset. Note that the velocity and time are given as non-dimensional quantities; (b) Two incommensurate frequencies in the Fourier power spectrum for the time series in Figure (a) indicate the onset of the quasiperiodic-like motion.

However, if the induced acceleration changes are longer (*t_w_* = 100) and high-frequent (*T* = 3), the quasiperiodic-like motion with modulated amplitude steps in. We interpret this as a transition to a new precursory creep regime, see Figure 3a. Quasiperiodicity on a torus is corroborated by the presence of two incommensurate frequencies in the corresponding Fourier power spectrum (Figure 3b).

**Figure 4:**
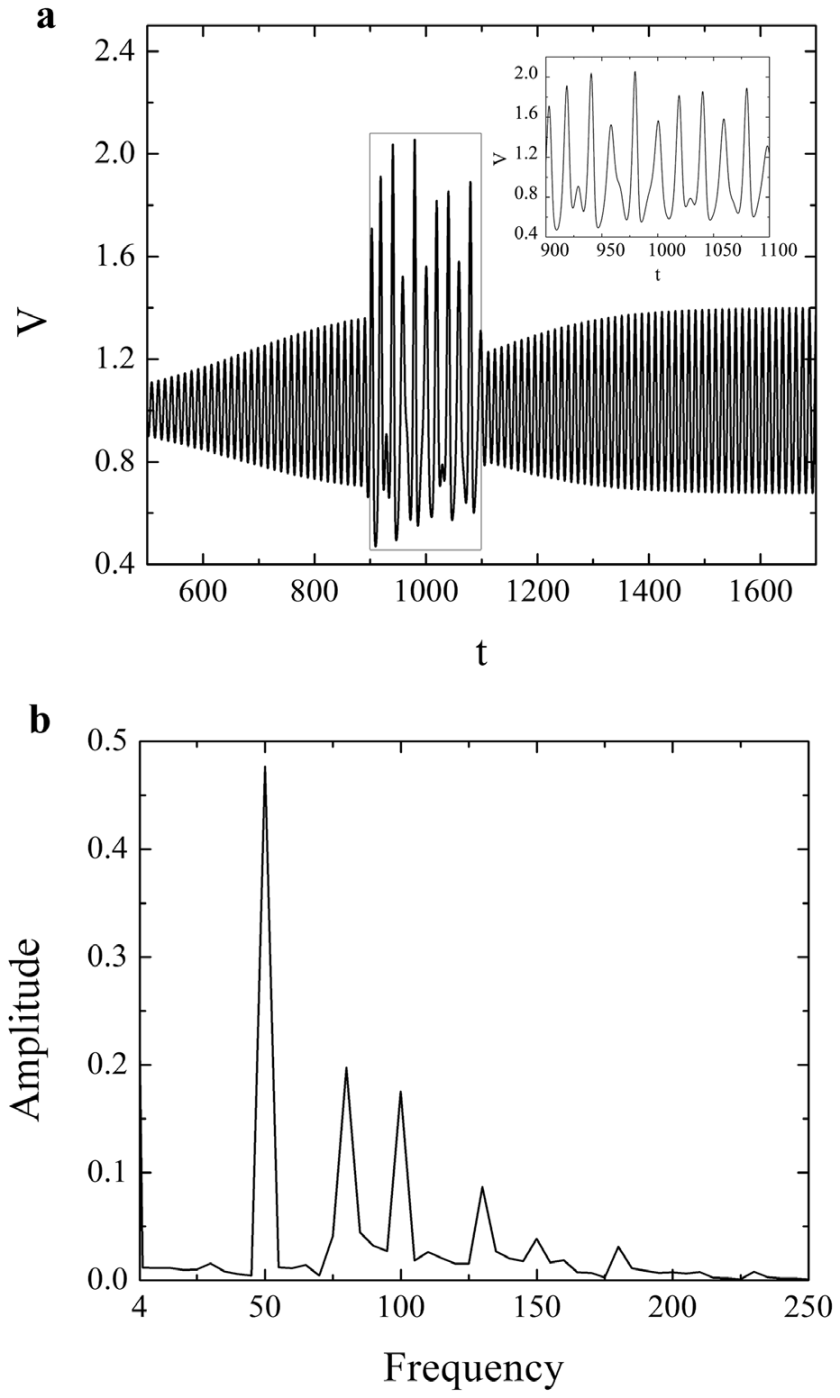
(a) Evolution of variable *V* (1) for *ε* = 0.3, *ξ* = 0.5 and *γ* = 0.8 and for low-frequent induced acceleration change (*T* = 20) with longer duration (*t_w_* = 100). The remaining parameters are set to: *A* = 0.6, *t_0_* = 1000 and *n* = 16. The rectangular frame indicates the irregular (chaos-like) transient part of the time series, which is shown enlarged in the inset. Note that the velocity and time are given as non-dimensional quantities; (b) Continuous broadband noise in Fourier power spectrum for time series in Figure (a) implies a possible chaos-like behavior of the system.

**Figure 5:**
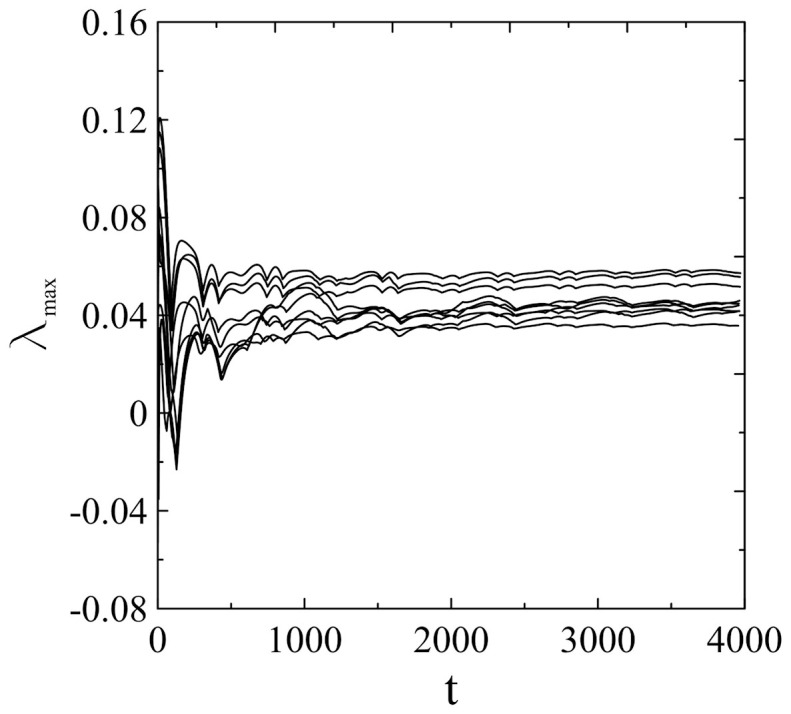
Calculation of the maximal Lyapunov exponent. The point that it is calculated for a transient irregular sequence requires one to perform additional averaging over a set of different initial conditions, whereby *θ*, *U*, *V* belong to the respective ranges 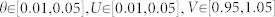. The results are obtained by adapting the method of Wolf et al.^26^. Maximal Lyapunov exponents are found to converge well to positive values in the range 3.6 − 5.7 × 10^−2^. Note that time *t* is expressed in the units of iteration steps.

If we now assume longer low-frequent changes (*t_w_* = 100 and *T* = 20), irregular oscillations are found, which correspond to transient seismic motion along the fault, see Figure 4a. Broadband noise in Fourier power spectrum indicates chaos-like behavior (Figure 4b), which is further verified by the positive value of maximal Lyapunov exponent for the different initial conditions (Figure 5).

**Figure 6:**
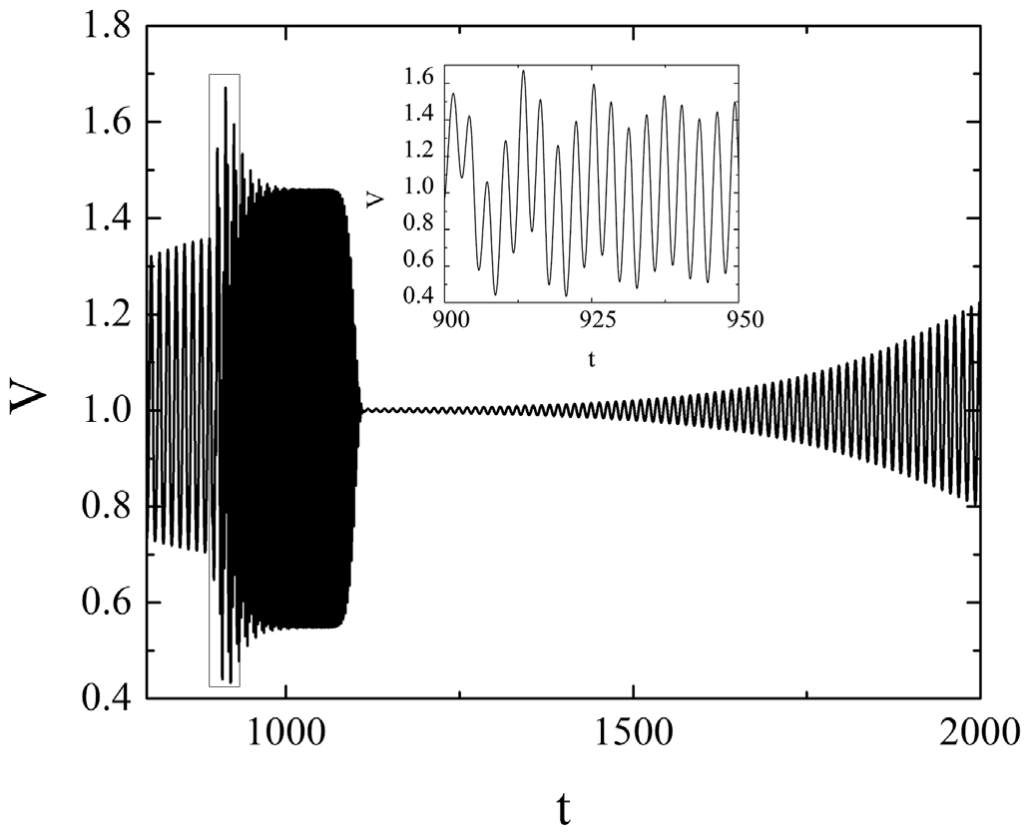
Temporal evolution of variable *V* for (*ε*, *ξ*, *γ*) = (0.3,0.5,0.8) when the system is subjected to a short-period acceleration change (*T* = 3). Other parameters are set to: *A* = 1.2, *t_w_* = 100, *t_0_* = 1000 and *n* = 16. The rectangular window indicates the time sequence with transient irregular motion, which is shown enlarged in the inset. Note that the velocity and time are expressed in nondimensional units.

Another interesting dynamical feature is seen if the amplitude of the induced change is varied during the inter-seismic creep regime. If the high-frequent changes of acceleration attain a larger amplitude (*A* = 1.2), only a short interval of quasiperiodic-like motion is observed, much shorter in comparison to the case shown in Figure 3 where *A* = 0.6. From Figure 6 one reads that the duration of the quasiperiodic-like motion is about four times shorter (*t* ≈ 50) compared to the case with the smaller amplitude in Figure 3 (*t* ≈ 200). Furthermore, apart from the short quasiperiodic-like motion, there is a temporary transition to post-seismic creep regime, with much slower convergence to the stable limit cycle. Nevertheless, if the low-frequent transient changes acquire the larger amplitude, say *A* = 1.2, no significant dynamical phenomena can be reported.

In case when the induced brief high-frequent acceleration changes are of low amplitude (*A* 0.3), the system (4) is found to responds with a sharp amplitude peak, after which it converges rapidly to a stable limit cycle. Similar effect is observed in case of low-frequent acceleration changes, when only small alterations of oscillation amplitude occur without any transition to another creep regime or to seismic motion.

In the last stage of the analysis, concerning the large values of perturbation amplitude, an effect of duration of transient change was examined by varying the parameter *t_w_*. It turns out that if a higher amplitude (*A* = 1.2) is assumed for both the high-frequent and the low-frequent transient, acting in inter-seismic creep regime, longer acceleration changes (*t_w_* = 800) further induce a switch to uniform motion, i.e. from inter-seismic to post-seismic creep regime, on a much longer time scales before system itself converges again to stable limit cycle. In case of post-seismic creep regime, long-term transients (*t_w_* = 800) of higher amplitude (*A* = 1.2) result only in a temporary transition to inter-seismic creep regime (i.e. oscillatory motion).

## Discussion

In the present paper, we have analyzed the effect of a transient periodic external force induced by a passing transient wave on the dynamics of the spring-slider model. The effect of a transient force is introduced to describe the dynamic triggering impact of nearby or distant earthquakes. The considered single-block model, incorporating the Dieterich-Ruina friction law between the block and the rough surface of the lower plate, is supposed to reproduce creep along the fault. The analysis of the original model (2) indicates that the transition from equilibrium state to stable periodic motion occurs via the direct supercritical Hopf bifurcation for certain values of the control parameters. The influence of a transient external force has been examined for two distinct states along the fault: uniform creep mode, represented by the steady state of the block, and the oscillatory creep mode, corresponding to the stable periodic motion of the block. These two states are interpreted to conform to the post-seismic and inter-seismic creep regimes of fault motion, respectively. The impact of the external force due to passing seismic waves is modeled through a change of block's acceleration, whose parameters are set with respect to the periodic oscillations of the unperturbed system (2) just above the bifurcation curve. These oscillations are characterized by the amplitude *A_0_* = 0.6 and the period *T_0_* ≈ 12. In this context, we have considered the short-period (*T* < 12) and long-period (*T* > 12) acceleration changes of different duration 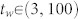. It is assumed that the brief short-period transient changes might correspond to closer, smaller earthquakes, while longer, low-frequent transients could represent the effect of larger, more remote earthquakes^13^.

Under the assumption that a passing seismic wave propagates along the fault, dynamics of system (4) reveals the following picture. If the induced acceleration changes are short, system (4) exhibits only the sharp and slightly higher amplitude response, with a slower convergence to an equilibrium state in the uniform creep mode, or a faster convergence to a stable limit cycle, when it comes to oscillatory creep mode. In case when the generated change is of longer duration (*t_w_* = 100), different dynamical features are observed, depending on the frequency of the perturbation of acceleration. A high-frequent acceleration changes in the inter-seismic creep-regime further lead to quasiperiodic-like dynamics, which is assumed to correspond to a precursory creep regime. However, long-period changes of acceleration in both post-seismic and inter-seismic creep regimes invoke irregular oscillations or chaos-like behavior, which can be treated as an onset of the seismic motion. Taking this into account, one may conclude that the longer low-frequent transients could excite a seismic motion even if the fault is in a stable uniform creep regime. Such type of triggering, analogous to the onset of earthquakes within the regions with low triggering threshold, has been observed in the case of series of earthquakes in Greece following the *M_w_*7.4 Izmit earthquake^6^. These findings are also consistent with the results of Brodsky and Prejean^27^, who determined that long-period waves are more effective at generating local seismicity than the short-period waves of comparable amplitude. This analysis covered the records of 12 regional and teleseismic events recorded at Long Valley Caldera.

If one assumes considerably longer acceleration changes (e.g. *t_w_* = 800), the only significant dynamical effect arises in the case of inter-seismic creep regime for high-amplitude transients. Apparently, it turns out that such long-term changes further induce a temporary transition from oscillatory to uniform motion, i.e. from an inter-seismic to post-seismic creep regime, after which the system converges to a stable limit cycle. A possible explanation of such a transition from oscillatory creep mode to uniform creep mode could be found in the properties of the fault gouge and the “maturity” of the fault. San Andreas fault system, for example, did not experience significant triggering by the Landers earthquake, due to its thicker and more deformed fault gouge that requires greater stress change to induce a rupture^7^.

The performed analysis has confirmed the occurrence of immediate or instantaneous dynamic triggering effect of a passing seismic wave, which has already been observed in situ following Landers^11^, Denali^12^ and Izmit earthquake^6^. A background mechanism for such an event is in relation with the immediate Coulomb-type failure^7^. In the present case, it is determined that the long-period transients, which are assumed to correspond to the effect of distant earthquakes, have the strongest impact on the fault motion, leading to irregular chaos-like behavior, i.e. to the onset of seismic motion. Nevertheless, the high-frequent transients, presumably originating from the nearby earthquakes, further induce a transition to quasiperiodic-like motion, which is recognized as a third, precursory creep regime that typically occurs just before the seismic motion commences. This effect is analogous to the dynamics of the spring-slider model with time delay, where the Ruelle-Takens-Newhouse route to chaos^28^ may be found.

Our analysis indicates that the amplitude of the induced acceleration change probably represents one of the most important influential parameters when evaluating the triggering effect of a passing transient wave. Apparently, the dynamics of system (4) under high-amplitude acceleration changes exhibits only the onset of periodic oscillatory motion of higher amplitude, after which the system converges to equilibrium state or a stable limit cycle. When such transients occur in the post-seismic regime, they further generate a short transition to inter-seismic regime, with slow convergence to an equilibrium state. Nevertheless, the low-amplitude variations have no significant impact on the dynamics of system (4). However, one should be aware of the fact that previous studies revealed that even very small dynamic changes (down to 3 × 10^−9^) could trigger faults that are sufficiently near failure^29^,^30^. Here we underline again that our model represents only the fault creeping that is relatively stable with respect to the short-lived perturbations in loading conditions and that the dynamics of this model does not exhibit transient or asymptotic instability without the external perturbation of sufficiently high amplitude.

Note that the performed analysis did not reveal the occurrence of a delayed triggering, which is typically observed under the real conditions in the Earth's crust. This is reminiscent to the earlier work of Gomberg et al.^13^,^31^, where it is indicated that, at least with respect to rate-and-state friction, the transient dynamic stresses could not explain the delay triggering. This is also consistent with Scholz^32^, who suggested that the direct effect of friction, as well as the finite size and duration of nucleation, prevent earthquakes from being triggered by the high-frequency stress oscillations, such as those generated by the passing seismic waves. Similar conclusions are also reached in Belardinelli et al.^16^, who found that a dynamic stress pulse is able to promote a nearly instantaneous failure, but cannot induce delayed triggering. In our setup, the main reason for the absence of delayed triggering lies in the properties of the chosen model itself, since it does not display irregular motion without the effect of external perturbation. All these arguments suggest that the delayed triggering requires an additional mechanism, such as the one that could lead to a time-dependent increase in the pore pressure. This way, the effective normal stress would be reduced, which is likely to eventually result in triggering of earthquake^7^,^11^.

One should point out that the presented analysis is non-dimensional, meaning that it only qualitatively captures the basic dynamical mechanism behind the triggering impact of the external signal to the fault motion. In this context, the applied model cannot be expected to capture the full complexity of seismic motion and the impact of the external signal. Further, it is not possible to make quantitative interpretations or predictions. However, what this model does provide is a framework for understanding how the transition between different creep regimes or triggered seismicity may be caused by seismic waves induced by near or distant earthquakes.

Future research could include the analysis of the stochastic effects of seismic noise on the dynamics of the presented model. Moreover, it would be interesting to examine the dual effect of both the additive and the multiplicative noise, with the included external signal, in order to verify whether some longer or even permanent instabilities are possible within the analyzed system. Incorporating another important natural ingredient, the seismic noise, in the analysis, would in qualitative terms make the model closer to the actual conditions observed in the Earth's crust.

## Methods

The system of first-order ordinary differential equations (2) has a unique stable stationary solution (*θ*, *U*, *V*) = (0, 0, 1), which corresponds to the steady sliding of the block. Hence, by this model, the fault is in a state of constant motion, which makes it more sensitive to the impact of dynamic triggering rather than the static stress changes. Under the real conditions in the Earth's crust, such a fault motion could correspond to some plate boundary segments, like an Eastern California shear zone, where the average repeat time of fault seismic motion is of the order of thousands of years^7^.

To analyze the dynamics of system (2) around the stationary solution (0,0,1), we numerically integrate the system (2) using the Runge-Kutta fourth order method for the different values of the parameter set *(ε, ξ, γ)*. The analysis on stability of the autonomous system (2) under variation of *ε* or *ξ* indicates a transition from equilibrium to periodic motion (obtained by increasing *ε above ε* = 0.27) and vice versa (obtained by reducing *ξ* below *ξ* = 0.49). At each instance, the parameters held constant attain values which admit the stable equilibrium (*ε* = 0.2, *ξ* = 0.5 and *γ* = 0.8). If both control parameters are varied in the *(ε, ξ)* parameter plane, the equilibrium goes unstable through the direct supercritical Hopf bifurcation, where a new stable limit cycle is created.

In the present paper, we make an interpolation that the different stages of block motion separated by the supercritical Hopf bifurcation curve correspond to the different creeping regimes along the fault. In particular, the uniform motion of the block is treated as a model of fault slip in the post-seismic creep regime (i.e. after-slip), just after the last seismic event has occurred. Nevertheless, the periodic oscillatory motion is considered as corresponding to the displacement along the fault in the inter-seismic creep regime. This refers to the more “mature” faults during the period of slow accumulation of elastic strain that coincides with the frictional locking of a fault between the earthquakes. Consistent with the two assumptions above and taking into account the absence of the coseismic stick-slip motion in the applied model, the periodic oscillations just above the Hopf bifurcation curve cannot be interpreted as an alternation between the slow interseismic phase and the coseismic stage^19^. On the contrary, the model (2) only describes the different stages of the creeping aseismic displacement along the fault, whereas the onset of transient quasiperiodic-like and irregular chaos-like motion occurs only under the effect of external perturbation. The two latter types of motion are assumed to correspond to the precursory creep regime and the seismic motion, respectively. In this context, the presented approach differs significantly from the standard seismological method, which assumes that the dynamic instability arises when the sliding velocity exceeds some pre-defined threshold value^33^,^34^,^35^,^36^. Our approach consists in analyzing the motion of a spring-slider model by the methods of nonlinear dynamics and chaos theory. The main aim is to understand the key mechanisms which govern the transition between the different creep regimes, with the ultimate goal to determine whether the onset of seismic motion is possible under the effect of external dynamic triggering.

As for the impact of a passing seismic wave, we have followed the general approach of Gomberg et al.^13^ and Perfettini et al.^14^, where a passing seismic wave is described by a simple function in the form of a sine wave scaled by an exponential pulse. Nonetheless, we do not consider explicitly the form of the ensuing seismic wave, but only its effects on the dynamics of acceleration. Hence, in contrast to previous studies^19^, one does not assume that the sliding acceleration is negligible, which implies a faster evolution of the fault in both the post-seismic and the inter-seismic creep regimes.

## Author Contributions

S.K., I.F., M.P., N.V. and K.T. designed and performed the research as well as wrote the paper.
